# Disease Prediction Models and Operational Readiness

**DOI:** 10.1371/journal.pone.0091989

**Published:** 2014-03-19

**Authors:** Courtney D. Corley, Laura L. Pullum, David M. Hartley, Corey Benedum, Christine Noonan, Peter M. Rabinowitz, Mary J. Lancaster

**Affiliations:** 1 Pacific Northwest National Laboratory, Richland, Washington, United States of America; 2 Oak Ridge National Laboratory, Oak Ridge, Tennessee, United States of America; 3 Georgetown University Medical Center, Washington, DC, United States of America; 4 Yale University School of Medicine, New Haven, Connecticut, United States of America; Université Catholique de Louvain, Belgium

## Abstract

The objective of this manuscript is to present a systematic review of biosurveillance models that operate on select agents and can forecast the occurrence of a disease event. We define a disease event to be a biological event with focus on the One Health paradigm. These events are characterized by evidence of infection and or disease condition. We reviewed models that attempted to predict a disease event, not merely its transmission dynamics and we considered models involving pathogens of concern as determined by the US National Select Agent Registry (as of June 2011). We searched commercial and government databases and harvested Google search results for eligible models, using terms and phrases provided by public health analysts relating to biosurveillance, remote sensing, risk assessments, spatial epidemiology, and ecological niche modeling. After removal of duplications and extraneous material, a core collection of 6,524 items was established, and these publications along with their abstracts are presented in a semantic wiki at http://BioCat.pnnl.gov. As a result, we systematically reviewed 44 papers, and the results are presented in this analysis. We identified 44 models, classified as one or more of the following: event prediction (4), spatial (26), ecological niche (28), diagnostic or clinical (6), spread or response (9), and reviews (3). The model parameters (e.g., etiology, climatic, spatial, cultural) and data sources (e.g., remote sensing, non-governmental organizations, expert opinion, epidemiological) were recorded and reviewed. A component of this review is the identification of verification and validation (V&V) methods applied to each model, if any V&V method was reported. All models were classified as either having undergone Some Verification or Validation method, or No Verification or Validation. We close by outlining an initial set of operational readiness level guidelines for disease prediction models based upon established Technology Readiness Level definitions.

## Introduction

A rich and diverse field of infectious disease modeling has emerged in the past 60 years and has advanced our understanding of population- and individual-level disease transmission dynamics, including risk factors, virulence, and spatio-temporal patterns of disease spread [1]–[4]. These modeling techniques span domains from biostatistical methods to massive agent-based, biophysical, ordinary differential equation (ODE), to ecological-niche models [5]–[8]. Diverse data sources are being integrated into these models as well, such as demographics, remotely sensed measurements and imaging, environmental measurements, and surrogate data such as news alerts and social media [9]–[11]. Moreover, nascent research is occurring at the omics-level to aid in forecasting future epidemics; such research includes phylogenetic techniques for predicting pathogen mutations, algorithms for microbial identification in next-generation technologies, meta-genomics, and multi-scale systems biology [12], [13]. Yet emerging infectious diseases continue to impact the health and economic security across the globe. There remains a gap in the sensitivity and specificity of these modeling forecasts designed not only to track infectious disease events but also predict disease occurrence [14]–[16]. For an example one needs to look no further than the 2009 H1N1 influenza pandemic. The latency between identification and characterization of the virus pathogenicity and transmissibility caused, perhaps, unnecessary mitigation measures such as school and business closures [17]–[19]. Moreover, there are strong indicators that dynamics and emergence of vector-borne diseases are in flux because of, among other factors, changes in land use, human behavior, and climate [20]–[22].

The goal of this systematic review was to identify areas for research to characterize the viability of biosurveillance models to provide operationally relevant information to decision makers about disease events. We define a disease event to be a biological event characterized by evidence of infection and or disease in humans, animals, and plants (i.e. the One Health paradigm). These disease events are neither mutually exclusive nor limited to the following examples for evidence of infection: person-to-person transmission (e.g., *Mycobacterium tuberculosis*), zoonoses (e.g., *Francisella tularensis*), food-borne pathogens (e.g., *Salmonella*), vector-borne pathogens (e.g., equine encephalitis virus), waterborne pathogens (e.g., *Vibrio cholerae*), airborne pathogens (e.g., influenza), veterinary pathogens (e.g., *Aphtae epizooticae*), and plant pathogens (e.g., soybean and wheat rusts). Examples for evidence of condition include accidental or deliberate events affecting air or water quality (e.g., volcanic ash, pesticide runoff), economically motivated adulteration of the food and pharmaceutical supply, and intentional exposure. In the context of this article, a biosurveillance model is broadly defined as an abstract computational, algorithmic, statistical, or mathematical representation that produces informative output related to event detection or event risk [23]. The model is formulated with a priori knowledge and may ingest, process, and analyze data. A biosurveillance model may be proactive or anticipatory (e.g., used to detect or forecast an event, respectively), it may assess risk, or it may be descriptive (e.g., used to understand the dynamics or drivers of an event) [23].

There also is a true lack of implementation of such models in routine surveillance and control activities; as a result there is not an active effort to build and improve capacity for such model implementation in the future [24]–[27]. When it comes to emerging infectious disease events, or the intentional or accidental release of a bioterrorism agent, most such pathogens are zoonotic (transmitted from animal to human) in origin [28]–[30]. Therefore, in assessing disease prediction models for biosurveillance preparedness, it is reasonable to include a focus on agents of zoonotic origin that could arise from wildlife or domestic animal populations or could affect such animal populations concurrently with human populations [31]. To date, the development of surveillance systems for tracking disease events in animals and humans have arisen largely in isolation, leading to calls for better integration of human and animal disease surveillance data streams [32], to better prepare for emerging and existing disease threats. Recent reports have shown some utility for such linkage [26], [33].

Two critical characteristics differentiate this work from other infectious disease modeling systematic reviews (e.g., [34]–[38]). First, we reviewed models that attempted to predict or forecast the disease event (not simply predict transmission dynamics). Second, we considered models involving pathogens of concern as determined by the U.S. National Select Agent Registry as of June 2011 (http://www.selectagents.gov).

## Methods

Subject matter experts were asked to supply keywords and phrases salient to the research topic. A sample of keywords and phrases used is shown in Table 1. Multiple searches were conducted in bibliographic databases covering the broad areas of medicine, physical and life sciences, the physical environment, government and security. There were no restrictions placed on publication date or language of publication. Abstracts and citations of journal articles, books, books in a series, book sections or chapters, edited books, theses and dissertations, conference proceedings and abstracts, and technical reports containing the keywords and phrases were reviewed. The publication date of search results returned are bound by the dates of coverage of each database and the date in which the search was performed, however all searching was completed by December 31, 2010. The databases queried resulted in 12,152 citations being collected. Irrelevant citations on the topic of sexually transmitted diseases, cancer and diabetes were retrieved. We de-duplicated and removed extraneous studies resulting in a collection of 6,503 publications. We also collected 13,767 web documents based on Google queries, often referred to as Google harvesting. We down selected the web documents for theses and dissertations, reducing this number to 21. Citations not relevant to the study of select agents, such as sexually transmitted diseases, cancer and diabetes, were identified and removed, leaving 6,524 documents. See Checklist S1 for a list of information sources used in this study.

**Table 1:** A Sampling of Keywords and Phrases.

Keywords and Phrases Used (not exhaustive):				
Biosurveillance	Disease forecast	Infectious disease surveillance	Remote sensing + disease forecast	Biosurveillance
Bioterror* and model	Disease outbreak origin	Pathogen detection	Spatial disease model	Bioterror* and model
CBRN model*	Epidemic model*	Population dynamic + outbreak	Vector-borne disease model	CBRN model*

+ Is used to link phrases or keywords with the Boolean operator “and”.

* Is used as truncation to search for words that begin with the same letters or to replace any number of characters.

Next, we filtered citations by hand based upon the definition of a biosurveillance model presented in the introduction and for select agents, which resulted in a 117 curated papers. Of these 117 papers, 54 were considered relevant to the study based on our selection criteria; however, 10 of these dealt purely with disease spread models, inactivation of bacteria, or the modeling of human immune system responses to pathogens. As a result, we systematically reviewed 44 papers and the results are presented in this analysis. See Figure S1 for a graphic summary of the data reduction methodology and Checklist S1 for the PRISMA guidelines used for the evaluation of the 44 papers. To enable real-time collaboration and sharing of the literature, the citations were exported to the Biosurveillance Model Catalog housed at http://BioCat.pnnl.gov.

The models in the selected publications were classified in the categories listed below. These categories are not mutually exclusive and publications involving multiple modeling and analytic approaches were assigned to multiple categories.

**Risk Assessment** models correlate risk factors for a specific location based upon weather and other covariates to calculate disease risk, similar to a forest fire warning. This type of model is commonly referred to as ecological niche modeling or disease risk mapping [39]–[41].

**Event Prediction** models will assign a probability for when and where the disease event is likely to occur based upon specific data sources and variables. The difference between event prediction and risk assessment is the end product of the model; in the former, the output is the location and a time period a disease outbreak will occur, while the risk assessment model provides the risk of an outbreak occurring under specified conditions [42]–[44].

**Spatial** models forecast the geographic spread of a disease after it occurs based upon the relationship between the outbreak and primarily geospatial factors. It should be noted that spatial models can be considered dynamical models in that they change in time, e.g., spatial patch models. [45]–[47].

**Dynamical** models examine how a specific disease moves through a population. These models may include parameters, such as movement restrictions, that have the effect of interventions on the severity of an epidemic or epizootic. These models may be used to predict and understand the dynamics of how a disease will spread through a naïve population or when the pathogenicity will change [48], [49].

**Event Detection** models attempt to identify outbreaks either through sentinel groups or through the collection of real-time diagnostic, clinical, or syndromic data and to detect spikes in signs, symptoms or syndromes that are indicative of an event (e.g., event-based biosurveillance) [50], [51].

The disease agents examined in this study were taken from the U.S. National Select Agent Registry and include human, plant, and animal pathogens. The agents described within these models are grouped non-exclusively by their mode of transmission: direct contact, vector-borne, water- or soil-borne and non-specific.

Next, we analyzed the data sources in order to find ways to improve operational use of biosurveillance models. These non-mutually exclusive data source categories were: “Epidemiological Data from the Same Location”; “Epidemiological Data from a Different Location”; “Governmental and Non-Governmental Organizations”; “Satellite (Remote Sensing)”; “Simulated”; “Laboratory Diagnostic”; “Expert Opinion”; and “Literature.” If a paper cited any form of literature that was not epidemiological, weather, or population data, it was categorized within the literature group. An example of this is references to the preferred natural habitat or survival requirements for a disease agent. Papers that cited epidemiological data from a location independent of the validation data were grouped “Epidemiological Data from a Different Location,” “Simulated Data,” and “Experimental Data.” “Expert Opinion” did not explicitly state from whom or what type of data was used. In addition to the model data sources, twelve non-mutually exclusive variable categories were identified to facilitate understanding of how these models could be used effectively by the research and operational communities. Models with variables describing location or distance and rainfall or temperature were categorized as “Geospatial” and “Climatic,” respectively. Models that took into account the epidemiological (population-level) characteristics of the disease were grouped together as “Epidemiological.” Variables that dealt specifically with the agent or etiology were categorized under “Etiology.” Population size, density, and other related variables were grouped into either “Affected Population” (i.e., the animal, plant, or human population affected by the disease) or “Vectors and Other Populations” (i.e., populations of the vector or any other population that may be considered within the model but that was not affected by the disease). Models that utilized remote sensing data such as the “normalized difference vegetation index” (NDVI), a measurement used to determine the amount of living green vegetation in a targeted area, were grouped within “Satellite (Remote Sensing).” “Agricultural” techniques, such as tillage systems, were also identified to be variables in some models as well as “Clinical” and “Temporal” variables. The final two variable types identified were “Topographic and Environmental,” such as altitude or forest type, and “Social, Cultural, and Behavioral,” which included religious affiliations and education.

There are many verification and validation (V&V) standards (e.g., ISO/IEC 15288-2008 [52], IEEE Std 1012-2012 [53], ISO/IEEE 12207 [54]) and definitions, including some that are specifically focused on modeling and simulation: NASA-STD-7009 [55], Verification, Validation, and Accreditation Recommended Practices Guide from the U.S. Department of Defense (U.S. DoD) Modeling & Simulation Coordination Office [56], U.S. Army TRADOC Reg 5-11 [57], U.S. Navy Best Practices Guide for Verification, Validation, and Accreditation of Legacy Modeling and Simulation [58], and U.S. DoD MIL-STD-3022 [59]. For instance, the U.S. DoD definition of *verification* for modeling and simulation is “the process of determining that a model implementation and its associated data accurately represent the developer's conceptual description and specifications”[56]. The US DoD definition of *validation* for modeling and simulation is “the process of determining the degree to which a model and its associated data provide an accurate representation of the real world from the perspective of the intended uses of the model”[56]. In the words of Boehm, verification answers the question “Did we build the system right?” and validation answers, “Did we build the right system?” [60]. Further, the “official certification that a model, simulation, or federation of models and simulations and its associated data is acceptable for use for a specific purpose” is its *accreditation* [56], which answers the question of whether the model/simulation is credible enough to be used.

All models were classified as either a) having undergone Some V&V method, or b) No V&V based only on the paper(s) cited for that model. Those models classified as having undergone Some V&V were further classified based upon the type of V&V method(s) applied to these models. The V&V method classifications used were “Statistical Verification”; “Sensitivity Analysis (verification)”; “Specificity and Sensitivity (verification)”; “Verification using Training Data”; “Validation using Temporally Independent Data”; and “Validation using Spatially and Temporally Independent Data.” In general, no conclusions on model credibility can be based on the types of V&V methods used, given that a) none of the papers were focused on the model V&V, and b) seldom are all aspects of V&V reported upon in the types of papers surveyed. The most frequently used verification method used is some form of statistical verification. It is important to note that verification methods do not necessarily imply that a model is correct. In this type of verification, methods such as Kappa (used to assess the degree to which two or more persons, examining the same data, agree on the assignment of data to categories), area under the receiving operating characteristic (ROC) curve, goodness of fit, and other statistical values are examined to help measure the ability of the model to accurately describe or predict the outbreak. Several models plotted observed data against predicted data as a V&V technique. This technique was further delineated, depending on whether the observed data were part the model's training data (verification), temporally independent of the training data (validation), or temporally and spatially independent of the training data (validation). The remaining models applied verification methods such as sensitivity analysis, which examined whether a model functioned as it was believed to when different values were input into important variables; or specificity and sensitivity metrics, which measure the ability to determine true positives and negatives. We acknowledge that not all of these V&V techniques are applicable to every model type. Also note that the use of a verification or validation method does not constitute complete verification or validation of the model. For instance, the IEEE standard for software verification and validation (IEEE Std 1012-2005) includes five V&V processes, supported by ten V&V activities, in turn implemented by 79 V&V tasks. To put this in perspective of the study, the V&V methods noted herein are at or below the level of task. Assessment of inherent biases present within these source documents and models reviewed is beyond the scope of this study.

## Results and Analysis

The publications' models were categorized as follows (see Table 2): event prediction (n = 4), spatial (n = 26), ecological niche (n = 28), diagnostic or clinical (n = 6), spread or response (n = 9), and reviews (n = 3). The event prediction type includes only four models—possibly explained by the difficulty in creating of a model that truly predicts disease events. In general, these models were applied to (or involved) small or special populations (e.g., populations with chronic diseases). According to Favier et al., the lack of prediction models could be addressed by taking a “toy model” and creating a predictive model [61]. If models that are similar to predictive models, such as risk assessment, could be modified into such, the number of predictive models could be increased.

**Table 2:** Citations categorized by model type.

Model Type	Citations	Total
Dynamical	[74]–[82]	9
Event Detection	[80], [83]–[87]	6
Event Prediction	[74], [88]–[90]	4
Review Articles	[91]–[93]	3*
Risk Assessment	[21], [62], [75]–[78], [88], [91], [94]–[107]	28
Spatial	[61], [74], [75], [78], [79], [83]–[85], [89], [94]–[99], [108], [109]	26

The categories are not mutually exclusive.

* The authors acknowledge others significant work in event-based biosurveillance, such as the G-7 Global Health Security Action Group [110], which is not cited in this table because of the selection criteria.

### Transmission Mode

The transmission modes of the models disease agent spanned the following: direct contact (n = 24), vector-borne (n = 15), water- or soil-borne (n = 7), and non-specific (n = 3); (see Table 3). Direct contact and vector-borne models accounted for approximately 84% of all of the evaluated models.

**Table 3:** The citations placed in each mode of transmission group.

Agent Mode of Transmission	Citations	Total
Direct Contact	[74], [75], [77]–[84], [88], [90], [93], [97], [99]–[101], [104], [105], [107], [108], [111]–[113]	24
Non-Specific	[86], [92], [114]	3
Vector-Borne	[61], [62], [76], [85], [87], [91], [94], [95], [97], [102]–[107], [109]	15
Water-, Soil-Borne	[21], [84], [89], [91], [96], [98]	7

If a model involved multiple agents in different categories, the paper was placed in multiple groups.

### Data Sources and Variables

The data sources (e.g., remote sensing, non-governmental organizations, expert opinion, epidemiological) and variable parameters (e.g., etiology, climatic, spatial, cultural) for each model were recorded and reviewed (see Table 4). The two categories that contained the most data sources were “Epidemiological Data from the Same Location” (n = 25), such as a previous outbreak, and data gathered from an organization, such as census data. Thirty-two models used some type of “Literature” (n = 14) an important fact is that the majority of data used in the models were scientifically measured.

**Table 4:** Citation categorized by Data Source.

Data Source	Citation	Total
Epidemiological Data Different Location	[79], [93]	2
Epidemiological Data Same Location	[21], [75], [77], [79], [81]–[83], [85], [87], [89], [90], [94]–[97], [99], [101], [102], [104], [105], [108], [111]–[113]	25
Expert Opinion	[93], [115]	2
Governmental or Non-Governmental Organization	[21], [74]–[76], [78], [80], [83], [84], [87]–[89], [93], [94], [96], [97], [99]–[102], [105], [106], [111], [113], [115]	25
Laboratory Diagnostic	[82], [84], [95]	3
Literature	[61], [62], [77], [82], [83], [86], [88], [90], [92], [94], [98], [103], [106], [109]	14
Satellite (Remote Sensing)	[21], [87], [88], [94], [95], [99], [102]–[105], [107], [115]	12
Simulated	[74], [82], [93], [108]	4

If a model utilized data from multiple categories, it was placed in each.

Categories of variables and parameters utilized in the models supplemented the data sources. The two largest groupings were “Geospatial” and “Climatic” variables. According to Eisen et al. [62], models that do not use epidemiological data produce results with lower confidence such that users may not trust the results or may not trust that the findings are relevant. Similarly, users may not have faith that models are structured in a biologically meaningful way if biologic or epidemiologic data do not appear in a model [63]. Nonetheless, before incorporating epidemiological data in disease event prediction models, further research is needed to determine whether such data will increase the model's robustness, sensitivity, and specificity. Factors such as accuracy and precision of epidemiological data will influence this analysis.

**Figure 1:**
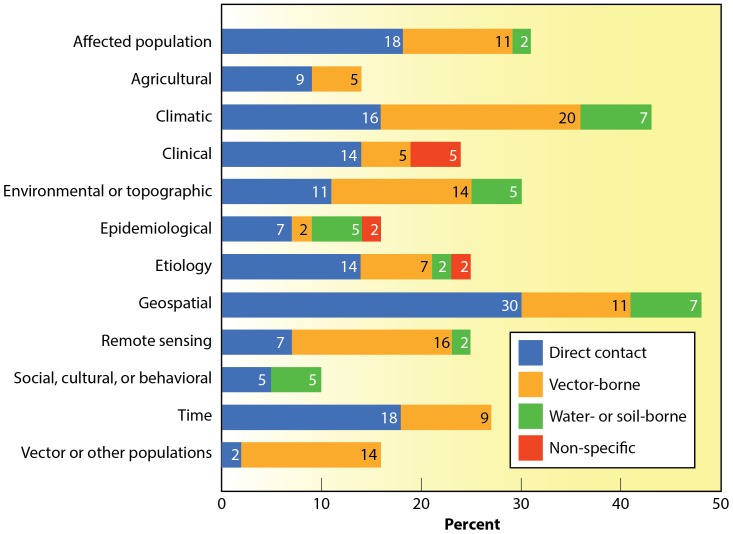
The Percentage of Citations Placed in Each Variable Group by Transmission Mode (if a model contained variables from multiple groups, it was placed in each respective group).

To better understand the relationship between the variables and the disease agent's mode of transmission, a graph (Figure 1) was created to show the distribution of different modes of transmission cited for each variable type used in the evaluated models. Table 5 shows the distribution of citations for each variable type. It was noted without surprise that, as more research was done on a mode of transmission, more variables were examined. Furthermore the variables, “Vectors or Other Populations” and “Social, Cultural, Behavioral” were underutilized in the evaluated models. This is unfortunate because these variables typically have a seasonal abundance pattern. Further, human socio-cultural behaviors greatly impact the interactions between human and vector populations and seasonal meteorological variation can strongly affect vector abundance and competence [64]. Relatively few disease prediction models were identified in which the causative agent was water- or soil-borne [20], [65].

**Table 5:** Citations Organized by Variable Group.

Variables Group	Citations	Total
Affected Population	[61], [75], [76], [79], [82]–[85], [88], [93], [95], [99], [101], [115]	14
Agricultural	[74], [79], [83], [90], [95]	5
Climatic	[21], [75], [76], [78], [80], [85], [87], [89]–[91], [94], [97], [98], [100], [102], [106], [107], [111], [115]	19
Clinical	[62], [76], [79], [92], [93], [101], [112]–[114]	10
Epidemiological	[61], [79], [81], [89], [92], [99], [111]	7
Etiology	[61], [62], [74], [77], [81], [82], [89], [111], [113], [114]	13
Geospatial	[62], [74], [75], [79], [80], [83], [84], [91], [93]–[96], [102], [104]–[106], [108], [109], [111], [115]	20
Remote Sensing	[21], [83], [87], [88], [94], [99], [102]–[105], [107]	11
Social, Cultural, Behavioral	[75], [93], [96], [98]	4
Time	[74], [82], [87], [94], [99], [100], [102], [108], [112], [113]	12
Topography or Environment	[78], [85], [88], [91], [95], [98]–[100], [102]–[106]	13
Vector or Other Populations	[61], [62], [75], [85], [87], [95], [115]	7

If a model contained variables from multiple groups, it was placed in each respective group.

### Verification and Validation Methods

The V&V methods applied to each model, if any, were also analyzed; see Table 6. Among the types of papers surveyed, few aspects of V&V are typically reported. The majority of models selected for this study were subjected some method of verification or validation. Publications on many applications of predictive models typically state statistical, sensitivity analysis, and training data test results. These are necessary, though insufficient methods to determine the credibility, verification or validation of a model. For instance, the IEEE standard for software verification and validation (IEEE Std 1012-2005) includes five V&V processes, supported by ten V&V activities, which are in turn implemented by 79 V&V tasks. To put this into perspective, the V&V methods noted herein are at or below the level of task. The papers reported the use of V&V methods for many models but not for others, and for the latter case it is unclear whether V&V methods were not used or merely unreported. Another positive observation is the significant use of real epidemiological data to examine aspects of model validity. Even though “Validation using Spatially and Temporally Independent Data” was used for one of the smallest sets of models, use of actual data versus predicted data for validation tests was reported for approximately 33% of the models. The reader is encouraged to understand that the use of a verification or validation method does not constitute complete verification or validation of the model [66]–[68].

**Table 6:** Grouping of Citations by Verification and Validation (V&V) Methods.

V&V Method	Citations	Total
No V&V	[61], [78], [86], [92], [109]	5
Sensitivity Analysis (verification)	[79]–[82], [94], [99], [112], [113]	8
Specificity and Sensitivity (verification)	[1], [75], [84], [95]	4
Statistical Verification	[21], [75], [77], [79], [82], [83], [88], [94]–[97], [99]–[106], [108], [115]	21
Validation using Spatially and Temporally Independent Data	[79], [90]	2
Validation using Temporally Independent Data	[84], [88], [97], [102], [103], [111]	6
Verification using Training Data	[21], [75], [81], [85], [89], [97], [104], [105], [107], [112], [115]	11

If a model used multiple methods for its verification or validation, it was categorized in each respective group.

### Operational Readiness

Given the importance of these models to national and international health security [69], we note the importance of a categorization scheme that defines a model's viability for use in an operational setting. To our knowledge, none exists, but below we illustrate one possibility, based upon the “technology readiness level” (TRL) originally defined by NASA [70], to evaluate the technology readiness of space development programs. Important to note: NASA TRL levels were not developed to cover modeling and simulation, much less biosurveillance models, so the definitions require modification. In the public health domain, TRLs can assist decision makers in understanding the operational readiness level, maturity and utility of a disease event or prediction model. Advantages of utilizing the TRL paradigm are that it can provide a common understanding of biosurveillance model maturity, inform risk management, support decision making concerning government funded research and technology investments, and support decisions concerning transition of technology. We also point out the characteristics of TRLs that may limit their utility, such as the operational readiness of a model does not necessarily fit with technology maturity (V&V), a mature disease prediction or forecasting model may possess a greater or lesser degree of readiness for use in a particular geographic region than one of lower maturity, and numerous additional factors must be considered, including the relevance of the models' operational environment, the cost, technological accessibility, sustainability, etc.

"Operational readiness" is a concept that is user and intended use dependent. A model that one user may consider ready may not suffice for readiness with another user. Different users have different needs according to their missions. For example, in the case of surveillance models, some will need to see everything reported by event-based surveillance systems (i.e., they are unconcerned with specificity but sensitivity is of high value to them), while other users may demand low false alarm rates (i.e., specificity is important for their needs) [71], [72]. The Operational Readiness Level rating of any given model will thus depend upon the diverse questions and purposes to which any given model is applied.

An initial scheme modifying these definitions is shown in Table 7. In such a scheme, the models would be characterized based on how the model was validated, what type of data was used to validate the model, and the validity of data used to create the model. The V&V of predictive models, regardless of realm of application, is an area that requires better definition and techniques. The results of model V&V can be used in the definition of model operational readiness; however the readiness level definitions must also be accompanied by data validation, uncertainty quantification, and model fitness for use evaluations, many of which are areas of active research [73].

**Table 7:** Initial Definitions of Operational Readiness Levels for Disease Prediction Models.

Level	Definition
1	Research only reported on observed information
2	A constructed model which has yet to be applied to data (or The model theory has been developed based on observed or hypothesized information)
3	The model has been created but has not been validated
4	The model has been verified and validated
5	The model has been demonstrated as useful but for only its original location (pathogen or population) and is still being updated to accommodate additional locations
6	The model has been demonstrated as useful in both its original location (pathogen or population) as well as an independent location (pathogen or population), but not all requisite locations
7…n	Further study is needed to explicitly delineate the criterion for all levels.

## Discussion

Our study was conducted to characterize published select-agent pathogen models that are capable of predicting disease events in order to determine opportunities for expanded research and to define operational readiness levels [38]. Out of an initial collection of 6,524 items 44 papers met inclusion criteria and were systematically reviewed. Models were classified as one or more of the following: event prediction, spatial, ecological niche, diagnostic or clinical, spread or response, and reviews. Model parameters (e.g., etiology, climatic, spatial, cultural), data sources (e.g., remote sensing, non-governmental organizations, expert opinion, epidemiological), and V&V methods applied to each model, if any, were identified. Moreover, an initial set of operational readiness level guidelines for disease prediction models, based upon established Technology Readiness Level definitions, were suggested.

In the majority of the models we examined, few aspects of V&V were reported. Although many models underwent some level of V&V, few if any demonstrated validation, and thus readiness, in a general sense that would find credibility with operational users. Such V&V is difficult to implement in general, for the reasons discussed in the previous section. However, if users are to apply the models and have confidence in model results, it is imperative to advance model V&V. Similarly, the suggested operational readiness level guidelines are meant to spur additional investigation, as our literature review uncovered no operational model readiness metrics. Given better definition of readiness levels, providing clear means to achieve upper operational readiness levels, and the ability to consistently assign confidence in readiness level assignment will lead to enhanced value to the decision makers. In order to test operational readiness levels, we suggest further development of the criteria and application of the levels to existing disease prediction models to evaluate their usefulness in an operational environment. Public health analysts and decision makers are in need of evidenced-based advice, and the value of operational readiness levels for the models on which they depend cannot be overstated.

## Supporting Information

Figure S1 **The PRISMA Flow Diagram.**
(PDF)

Table S1 **Information sources used in biosurveillance study.**
(DOCX)

Checklist S1 **The PRISMA Checklist.**
(DOC)
