# Supplementary figures and images for: Disease Prediction Models and Operational Readiness
Source: PLoS One. 2014 Mar 19;9(3):e91989. doi: 10.1371/journal.pone.0091989 (PMC3960139; doi:10.1371/journal.pone.0091989)

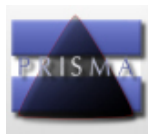

## PRISMA 2009 Flow Diagram

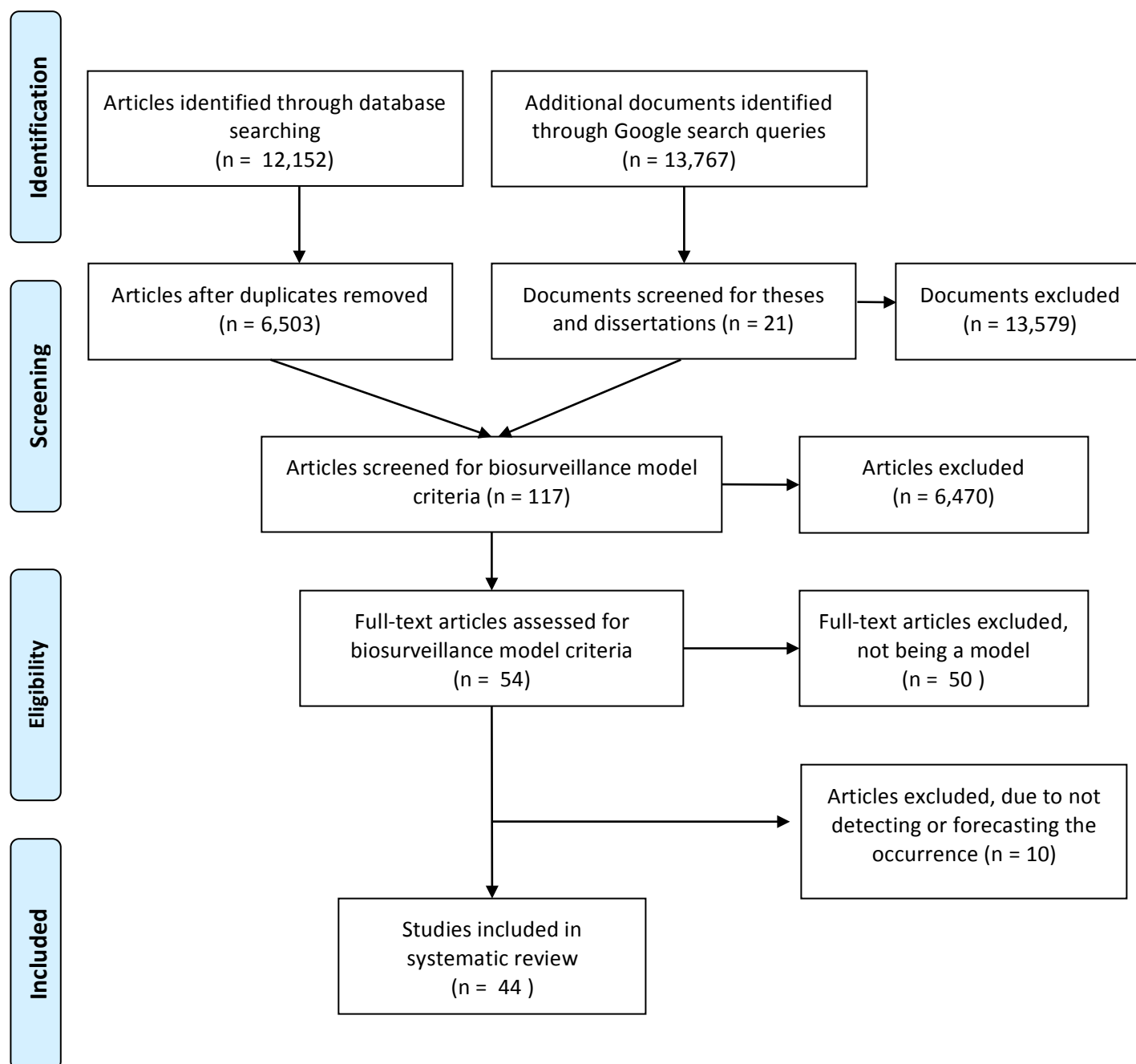

Supplement: Figure S1 — The PRISMA Flow Diagram. (PDF) [file pone.0091989.s001.pdf]
