# Supplementary material for: Disease Prediction Models and Operational Readiness
Source: PLoS One. 2014 Mar 19;9(3):e91989. doi: 10.1371/journal.pone.0091989 (PMC3960139; doi:10.1371/journal.pone.0091989)
Supplement: Table S1 — Information sources used in biosurveillance study. (DOCX) [file pone.0091989.s002.docx]

**Supporting Information**

The databases in Table S2 were readily available to the information analysis staff through Pacific Northwest National Laboratory library subscriptions and through the restricted access Open Source Center ([https://www.opensource.gov](https://www.opensource.gov/)). Databases selected for this study are international in scope and ensure we investigate relevant studies that are global and multidisciplinary, thereby eliminating the risk of bias from author subject matter expertise and a Western perspective. However, we are bound by the institutional subscriptions available.

| **Database** | **Dates of Coverage** | **Type of Database** |
| --- | --- | --- |
| Academic Search Complete | 1887-present | Subscription |
| ASFA: Aquatic Sciences and Fisheries Abstracts | 1971-present | Subscription |
| BioOne Abstracts and Indexes | 1998-present | Subscription |
| Compendex thru Engineering Village | 1884-present | Subscription |
| DTIC (private version) | Variable by agency | Registration Required |
| Environment Index | 1888-present | Subscription |
| GeoRef and GeoRef in Process | 1693-present | Subscription |
| GreenFILE | 1913-present | Publicly Available / Open Access |
| Google | Variable by website | Publicly Available / Open Access |
| Google News | Variable by source | Publicly Available / Open Access |
| Google Scholar | Variable by source | Publicly Available / Open Access |
| Homeland Security Digital Library | Varies by agency | Subscription |
| INSPEC thru Engineering Village | 1896-present | Subscription |
| International Security & Counter Terrorism Reference Center | 1921-present | Subscription |
| MEDLINE (through Web of Knowledge portal) | 1950-present | Subscription |
| Military & Government Collection | 1901-present | Subscription |
| NTIS thru Engineering Village | 1899-present | Subscription |
| Oceanic Abstracts | 1981-present | Subscription |
| Pollution Abstracts | 1981-present | Subscription |
| Scirus | 1900-present | Publicly Available / Open Access |
| Scopus | 1823-present | Subscription |
| Toxicology Abstracts | 1981-present | Subscription |
| Water Resources Abstracts | 1981-present | Subscription |
| Web of Science (Science edition), Conference Proceedings Citation Index (Science; Social Sciences & Humanities) | WoS, 1900-present  CPCI, 1990-present | Subscription |

The publication date of search results returned are bound by the dates of coverage of each database and the date in which the search was performed, however all searching was completed by December 31, 2010. The databases in Supplemental Table 1 were queried which resulted in over 12,152 citations being collected. An example search strategy for Web of Science (Science edition) plus Conference Proceedings Citation Index is as follows – No limits on timespan, Topic search, *"vector borne" disease surveillance dengue*.

Many abstracting and indexing databases have very broad coverage, which results in individual databases duplicating the content found in others. Duplicated bibliographic records were identified within the EndNote library and removed. After duplicate entries were removed from the results, the citations were reviewed to meet minimum citation requirements. Base data elements include those citation components deemed necessary for an end user to be able to use the citation to acquire the document from a library if so desired (e.g., authors, article name, journal name). If a citation did not contain the required elements, the source database was consulted to retrieve the additional information and was manually corrected.
